# Baseline characteristics and outcomes of the main perpetrator programme within the Hampshire Domestic Abuse Prevention Partnership, UK: A mixed methods study

**DOI:** 10.1371/journal.pone.0218408

**Published:** 2019-07-03

**Authors:** Sara Afshar Morgan, Beth Mary Stevens McCausland, Julie Parkes

**Affiliations:** 1 Department of Primary Care and Population Sciences, Faculty of Medicine, University of Southampton, Southampton, United Kingdom; 2 Department of Clinical and Experimental Sciences, Faculty of Medicine, University of Southampton, Southampton, United Kingdom; Queen's University at Kingston, CANADA

## Abstract

Effective perpetrator programmes should be embedded within a community response, engage all types of perpetrators and involve an educational approach that integrates the survivor’s voice. The Domestic Abuse Prevention Partnership (DAPP) is a transformative partnership based in the UK that aims to provide an integrated approach for perpetrators and survivors. This pragmatic mixed methods study was conducted to examine the baseline characteristics and individual outcomes of the main perpetrator programme within the DAPP. Applying a triangulation design, routine police re-offending aggregated data, pre- and post- perpetrator programme questionnaires, in-depth interviews with survivors, and focus-group discussions with perpetrators (clients) were integrated. Statistical analysis and thematic analysis were applied to quantitative and qualitative data, respectively. The majority of clients (47%) referred through the DAPP (n = 228) described *wanting to make their relationship better* as the main reason for engaging with the main perpetrators programme. Post-perpetrator programme questionnaires identified positive changes in both emotional behaviours and physical behaviours amongst clients, which were also supported by examples of improved relationships with their children described in survivor interviews. Three themes were described: first, *making positive progress;* second, *impact of the children’s module;* and *concerns around sustaining new behaviours*. Over the monitoring period, 1 in 5 clients were either suspected or convicted of domestic abuse crimes following the programme. This suggests that further maintenance of positive behaviours and reinforcements are required for some clients. Given that clients felt children were a strong motivating factor for completing a programme, it seemed paradoxical that no specialist services were made available for them. Future reiterations of the DAPP model should at least address how best to work with children in families where domestic abuse occurs.

## Introduction

National statistics for England and Wales estimate that 6.0% of adults aged 16–59 experienced domestic abuse in the last year, with a higher proportion experienced by women (7.9%) versus men (4.2%) [1]. These estimates are based on the latest UK government’s definition of domestic abuse, as *‘any incident or pattern of in*cid*ents of controlling*, *coercive*, *threatening behaviour*, *violence or abuse*, *between those aged 16 or over who are*, *or have been*, *intimate partners or family members regardless of gender or sexuality’* [2]. Domestic abuse has wider cost implications for the health and social care system, with direct medical and mental healthcare costs approximating £1730 million per annum in the UK [3, 4]. Intimate partner violence is particularly prevalent among both survivors and perpetrators receiving mental health services. Although the relationship is likely bi-directional, domestic abuse is associated with mental health problems, including depression and post-traumatic stress disorder (PTSD) [3, 5, 6].

Given that the majority of domestic abuse survivors are women, the development and implementation of perpetrator programmes often take a gendered view by examining the role, and influence, of power and control within the relationship. Most domestic violence perpetrator programmes (DVPPs) are therefore embedded in an understanding of heterosexual relationships; apply cognitive behavioural, pro-feminist or psychodynamic treatments; whilst 50% reportedly use a combination of multiple treatment types [7]. Over the years, DVPPs have developed through a process of critical reflection and engagement using the National Practitioners Network (NPN), as well as from listening and responding to perpetrators [8].

Voluntary community based programmes work with perpetrators of domestic abuse referred from community services, such as Children’s Services and the family courts. Such programmes often involve multiple agencies, meaning that there is increased opportunity for inefficiency, duplication and poor decision making [9]. Many community agencies, with a focus on survivors, also lack the adequate knowledge and competency of working with perpetrators. To circumvent some of the issues around dealing with perpetrators, voluntary community based programmes may use co-location, whereby referral agencies are supported through additional training and engagement. Given the complexity of embedding DVPPs within the community, and concerns around quality and efficiency, there is particular interest in modelling a multi-agency approach and evaluating the process of undertaking DVPPs, in addition to reporting the outcomes of such programmes [10].

The effectiveness of perpetrator programmes has long been debated. However, it is difficult to estimate the effectiveness of these programmes, primarily due to the difficulties arising from study methodology. Limitations in study design include small sample sizes, low response rates during follow-up, and no equivalent comparison or control group [11]. However, evidence suggests that programmes embedded within a coordinated community response, that aim to identify, treat and retain perpetrators, lead to the most positive response in terms of re-assault prevention [12].

### The Domestic Abuse Prevention Partnership (DAPP)

The DAPP is a multi-agency initiative based in Hampshire, UK led by a third sector organisation. The DAPP is a novel community based partnership that provides a tiered, flexible and needs-led approach; bridging specialist services for domestic abuse, with a focus on working directly with perpetrators. The DAPP is considered novel as it provides a voluntary programme (i.e. not court-mandated) for perpetrators and ensures that survivors are offered support at the same time. Furthermore, the DAPP provides one to one support for perpetrators whose lives are considered too chaotic to manage group work; in addition to the psycho-educational support offered through the one to one work, such individuals may be given additional support to find housing or employment, for example.

Under the DAPP, perpetrators (clients) engage with a 20-week programme, named the RADAR/ADAPT programme, consisting of modular group work. The modules include awareness raising, relationship dynamics, children and domestic abuse and parenting.

The aim of this study was to add to the limited evidence around community perpetrator programmes, and what they achieve, by evaluating motivations for engagement, type of abuse committed, as well as behavioural change outcomes following the 20-week programme, using data from both client and survivor.

## Methods

This was a mixed methods study with a triangulation design model, which aims to examine the effectiveness of the DAPP model by simultaneously triangulating both quantitative and qualitative data [13].

### Police data on reoffending (Record Management System)

Police reoffending data was used as a proxy indicator of harm caused to the partners, or former partners, by those who had attended the main perpetrator programme within the DAPP. Hampshire Constabulary provided aggregated police monitoring data on 80 clients who completed the programme between 01 April 2016 and 30 September 2017. The data included the total number of clients linked to any crime, as well as domestic-abuse related crime, either suspected or convicted.

### IMPACT monitoring (Pre- and post-programme questionnaire)

The IMPACT monitoring toolkit was developed by the Work with Perpetrators European Network to harmonise and enhance the monitoring and evaluation of work with perpetrators across Europe [14]. The IMPACT toolkit consists of a questionnaire that examines, through self-report, perpetrator behaviour (towards their partner or former partner) including type of abuse; their well-being; the impact on children; and their current relationship with their partner. To monitor progress through the main perpetrator programme offered through the DAPP, clients filled out the IMPACT questionnaire before (T0), and after, the main programme (T3).

### In-depth interviews

The qualitative methods included focus group discussions and in-depth interviews. The in-depth interview was chosen to explore outcomes for survivors, whose partner (or former partner) had engaged with the main programme. One-to-one interviews were considered the most appropriate means for exploring personal experiences and perceptions, as compared to any other qualitative methods. A support worker from the third sector organisation contacted and identified survivors who were willing to be interviewed. The majority of survivors did not have regular contact with their perpetrator and therefore could not provide accounts of the programme. Based on the difficulties of consenting survivors to be interviewed about their partner’s (or former partners) engagement with the programme, and the limitations in numbers, a maximum of 10 participants were to be recruited. The inclusion criteria included: survivors that acknowledged incidence of abuse through case reporting; survivors that were currently in contact with staff at the third sector organisation; and survivors that expressed willingness for feedback regarding clients’ progress through the programme.

### Focus group discussions

Focus group discussions were considered as the most appropriate way to draw on the experiences and perceptions of the majority of clients–as their experiences of the RADAR/ADAPT programme were constructed as a group. Due to the nature of running a 20 week programme, it was not possible to choose groups at random, as selection occurred during a short research period window of three months, when only a small number of groups were running. Based on feasibility, a maximum of two focus groups were selected, and took place before, or after, the planned programme session.

### Interview protocols

All in-depth interviews were carried out by SM, and the focus group discussions by both SM and BM. Whilst all participants engaged within focus group discussions were male, it is also necessary to acknowledge that both SM and BM are female researchers. A semi-structured topic guide was developed based on the literature, with both structure and flexibility, so that the order in which the topics were discussed followed a natural course. All interviews took place in a private location without any outside disturbances. Consent was required from each participant before the start of the interview. The recorded interviews were later transcribed.

### Data analysis

For quantitative data, statistical analysis was undertaken using Stata version. 14. For both IMPACT and police data, which were based on individual data, complete case analysis was used. Using the IMPACT data, descriptive statistics were used to summarise the client’s individual variables. Clients were asked about their behaviours reporting on a Likert scale: *never*, *sometimes* or *often*. There were a total of 14 descriptive questions of physical behaviour; 11 of emotional behaviour; and 7 of sexual behaviour. These responses were recoded into binary codes: 0 –never; and 1 to include those that responded either *sometimes* or *often*. These were then used to produce an overall continuous score for each behavioural type (emotional, physical, sexual) and are presented as prevalence (%).

To examine change over time, individuals were matched at T0 and T3, and behaviours were compared. Reoffending rates were determined using aggregated police data for those completing the programme over one year; showing the total crimes committed (as suspect or convicted) amongst completers, the proportion of total crimes that related specifically to domestic abuse, and the total number of clients (completers) that were implicated.

For qualitative data, the transcripts were exported into NVivo version 10, a qualitative data management software package. Using the thematic approach outlined by Braun and Clark, a six phase process was undertaken; in which patterns were identified, analysed and reported within the dataset [15]. Furthermore, negative case analysis was undertaken to look for patterns that contradicted patterns in the dataset.

### Research ethics

Ethical permissions were granted by the University of Southampton Ethical Committee (ID: 26211). Upholding ethics, good governance and quality in practice was central to all processes. These include the moral principles guided by four main principles of bioethics–as suggested by Beauchamp and Childress [16]. The principles are autonomy (informed consent), non-maleficence (do no harm), positive beneficence (benefits of research outweigh the risks) and justice (research strategies and procedures are just and fair). The social workers consented for routine data, from both clients and survivors, to be shared for the purposes of the evaluation by using a consent tick box on an assessment form. This routine data was shared for the research purposes as wholly anonymised data. During the evaluation, analysis, and write-up, no data was linked to the names of participants. For qualitative interviews, formal informed consent was obtained by researchers (SM, BM) for each participant. For survivors, the research interviews were conducted by phone for ease and privacy; verbal consent was obtained and audio-recorded. For clients, written consent forms were obtained. The qualitative quotes have been pseudonymised in this written report. All interview materials were approved by the Ethical Committee.

## Results

### IMPACT questionnaire

Between April 2016 and November 2017, 228 individuals completed the IMPACT questionnaire at baseline (T0) before the start of the programme. Over the same period 80 individuals completed the programme. The remaining 148 either failed to start or failed to complete the programme. The following Tables 1 and 2 show the reasons for coming to the programme at T0.

**Table 1:** Reasons for coming to the programme (self-report), using the IMPACT questionnaire (n = 228).

Main reasons for coming to the programme	Frequency, n	Prevalence, %
Reason: I have to come as part of my criminal court sentence or bail or parole conditions	1	0.4
Reason: I have to come because the family court told me to (voluntary basis)	10	4.4
Reason: I have to come because the child protection services told me to (voluntary basis)	54	23.7
Reason: I don’t want to go back to prison again	2	0.9
Reason: I want to be a better parent to my children	13	5.7
Reason: I want to stop using abusive behaviour	7	3.1
Reason: I don’t want my partner to be afraid of me	3	1.3
Reason: I don’t want my children be afraid of me	6	2.6
Reason: I want my partner/ex to feel safe around me	15	6.6
Reason: Social worker told me to	1	0.4
Reason: Solicitor told me to	1	0.4
Reason: I want my relationship to be better	107	46.9
Reasons: Quote given **	8	3.5
Total	**228**	**100.0**

**Note: Examples of quotes

‘Understand myself and learn more about self-control.’ ‘I want to be a better person and help others.’ ‘I want to see my son.’ ‘I want to do the best I can for my daughter.’

Approximately 47% of clients indicated that the main reason for coming to programme was to make their relationship better, whereas approximately 24% of clients came because child protection services had told them to.

**Table 2:** Relationship status of those assessed before the start of the programme (self-report, using the Impact questionnaire (n = 228).

RELATIONSHIP STATUS	Frequency, n	Prevalence, %
Together and living together	62	27.2
Together but living apart	55	24.1
In the process of splitting up	11	4.8
The relationship has ended and we are living apart	77	33.8
I am not sure	12	5.3
Something else–please say:*	11	4.8
Not answered	0	0
**Total**	**228**	**100**

*Note: Examples of quotes

‘Not together yet but need to do some hard work first. We are going to give a try one more time.’ ‘Have an injunction out against me.’ ‘Friend. Divorced. Living apart.’

The highest proportion of clients were no longer with their partners (33.8%), whereas approximately 1 in 4 individuals were still in a relationship with their partners and living together.

**Fig 1:**
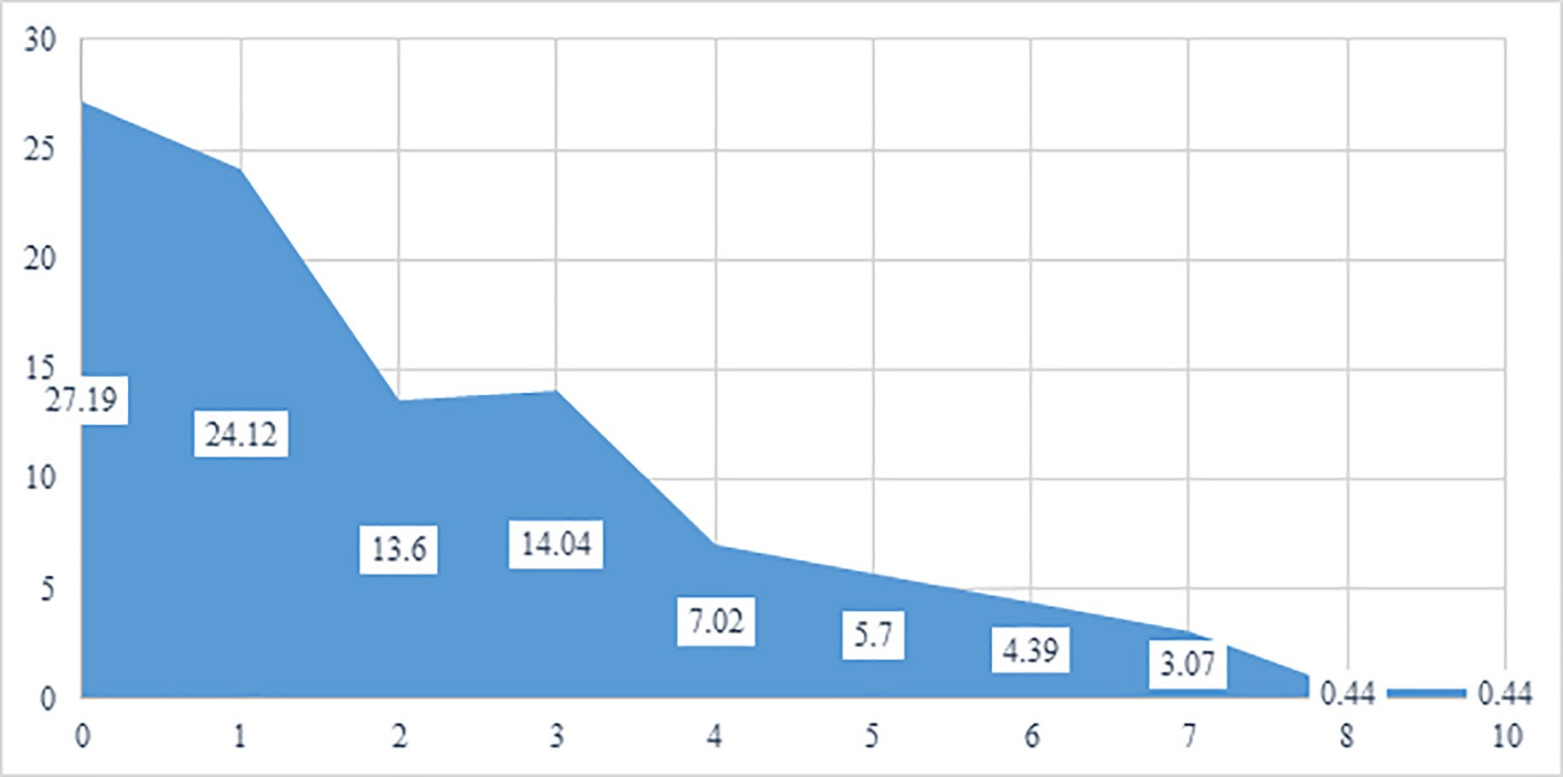
Emotional behaviour score (related to domestic abuse) of clients assessed at baseline, shows prevalence (%) based on N = 228. **Note: A total of 11 questions were used to produce this figure on the emotional behaviour of clients assessed at baseline:** 1) Emotional behaviour: Isolated from friends or family WITHIN the last 12 months; 2) Emotional behaviour: Told partner what to do/not do, where to go/not go, who to see/not see WITHIN the last 12 months; 3) Emotional behaviour: Made partner feel she had to ask permission to do certain things such as going out, seeing friends, etc. (above and beyond being polite) WITHIN the last 12 months; 4) Emotional behaviour: Threats to hurt the children WITHIN the last 12 months; 5) Emotional behaviour: Made them feel afraid by things you did/said WITHIN the last 12 months; 6) Emotional behaviour: Prevented partner/ex from leaving home WITHIN the last 12 months; 7) Emotional behaviour: Controlled the family money WITHIN the last 12 months; 8) Emotional behaviour: Threats to hurt partner/ex WITHIN the last 12 months; 9) Emotional behaviour: Extreme jealousy or possessiveness WITHIN the last 12 months; 10) Emotional behaviour: Told partner/ex what to wear or not to wear or how to do hair/makeup WITHIN the last 12 months; 11) Emotional behaviour: Humiliated/embarrassed partner/ex in front of others WITHIN the last 12 months.

As shown in Fig 1, 7% of clients exhibited none of the total eleven emotional behaviours listed. However, 24% exhibited only 1 behaviour and approximately 20% exhibited 4 or more of the total emotional behaviours.

**Fig 2:**
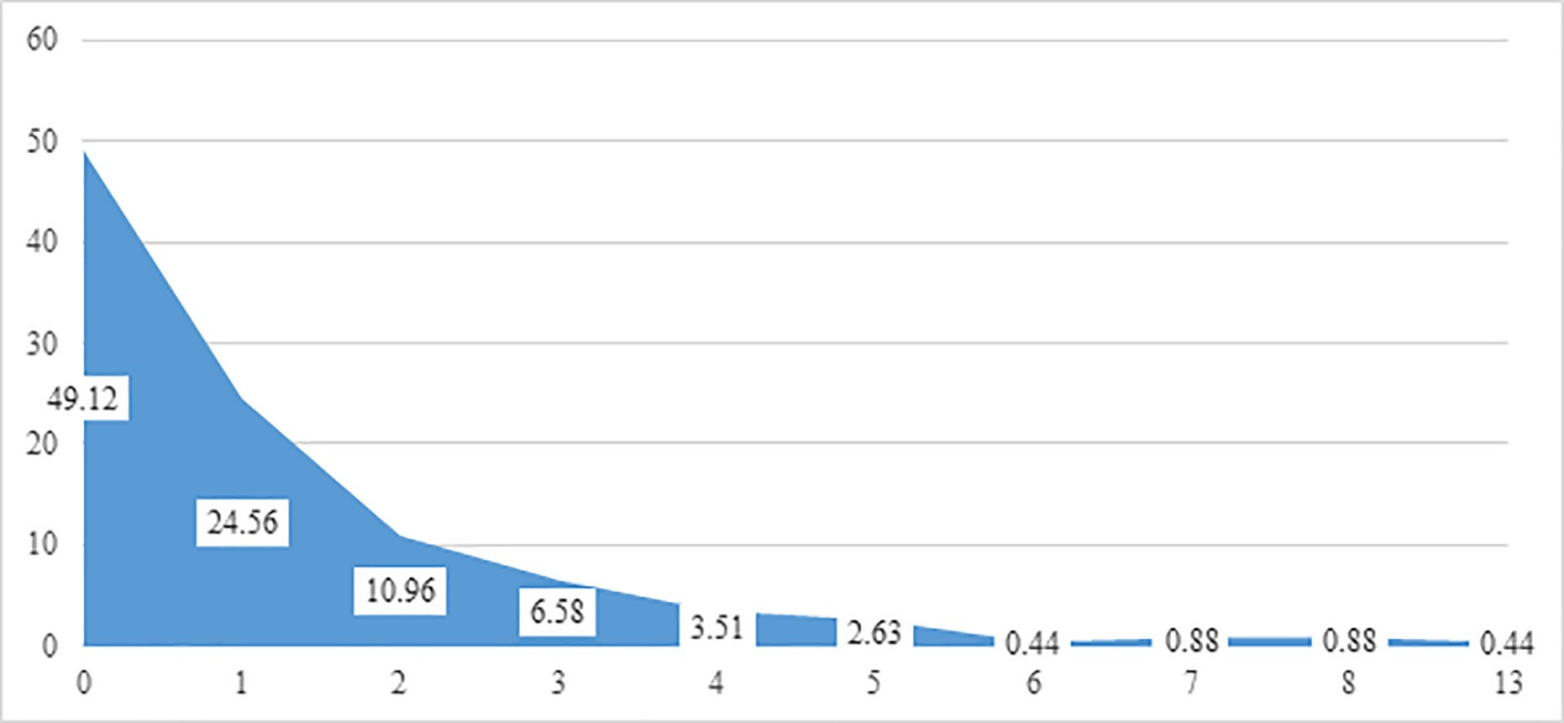
Physical behaviour score (related to domestic abuse) of clients assessed at baseline, shows prevalence (%) based on N = 228. **Note: A total of 14 questions were used to produce this figure on the physical behaviour of clients assessed at baseline:** 1) Physical behaviour: Slapped / pushed / shoved WITHIN the last 12 months; 2) Physical behaviour: Kicked / punched WITHIN the last 12 months; 3) Physical behaviour: Beaten up WITHIN the last 12 months; 4) Physical behaviour: Burned WITHIN the last 12 months; 5) Physical behaviour: Bitten WITHIN the last 12 months; 6) Physical behaviour: Restrained/held down/tied up WITHIN the last 12 months; 7) Physical behaviour: Put your hands on her throat or face (trying to choke or strangle or suffocate) WITHIN the last 12 months; 8) Physical behaviour: Physically threatened WITHIN the last 12 months; 9) Physical behaviour: Hit with object or weapon WITHIN the last 12 months; 10) Physical behaviour: Threatened with object/weapon WITHIN the last 12 months; 11) Physical behaviour: Threatened to kill her WITHIN the last 12 months; 12) Physical behaviour: Prevented her getting help for injuries WITHIN the last 12 months; 13) Physical behaviour: Stalked/followed/harassed her WITHIN the last 12 months; 14) Physical behaviour: Locked her in house or room WITHIN the last 12 months.

As shown in Fig 2, 49% of clients exhibited none of the total 14 physical behaviours listed. By contrast approximately 25% of clients exhibited 1 physical behaviour of the total listed.

**Fig 3:**
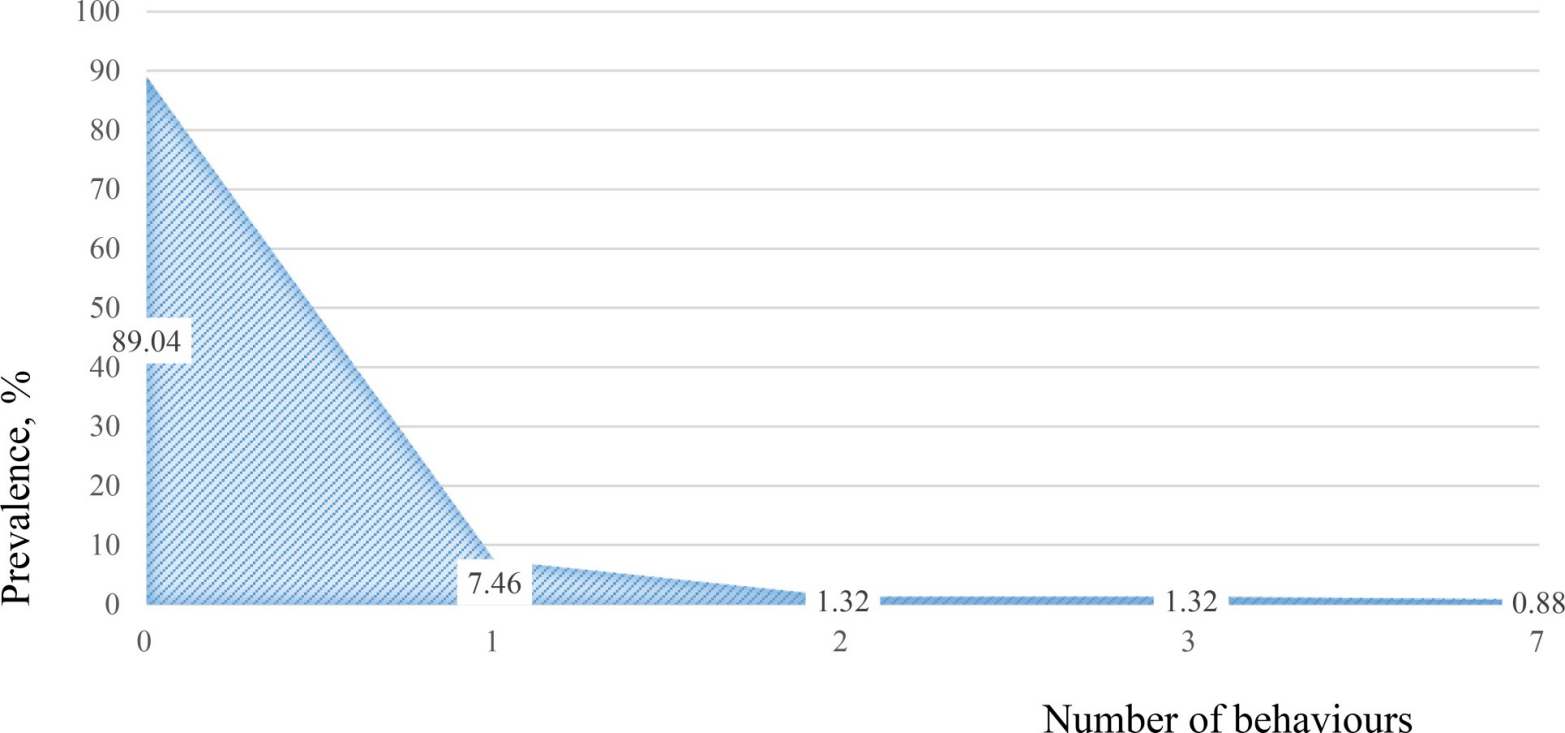
Sexual behaviour score (related to domestic abuse) of clients assessed at baseline, shows prevalence (%) based on N = 228. **Note: A total of 7 questions were used to produce this figure on the sexual behaviour of clients assessed at baseline** 1) Sexual behaviour: Touched in way which caused her fear/alarm/distress WITHIN the last 12 months; 2) Sexual behaviour: Forced her into doing something sexual she didn’t want to WITHIN the last 12 months; 3) Sexual behaviour: Hurt her during sex WITHIN the last 12 months; 4) Sexual behaviour: Disrespected boundaries or safe words WITHIN the last 12 months; 5) Sexual behaviour: Made her have sex when she didn’t want to or didn’t stop when she wanted to WITHIN the last 12 months; 6) Sexual behaviour: Sexually assaulted/abused her in any way WITHIN the last 12 months; 7) Sexual behaviour: Threats to sexual assault/abuse her WITHIN the last 12 months.

As shown in Fig 3, approximately 89% of clients reported exhibiting none of the sexual behaviours out of the list of 7; and only 11% reported exhibiting one or more sexual behaviours.

**Fig 4:**
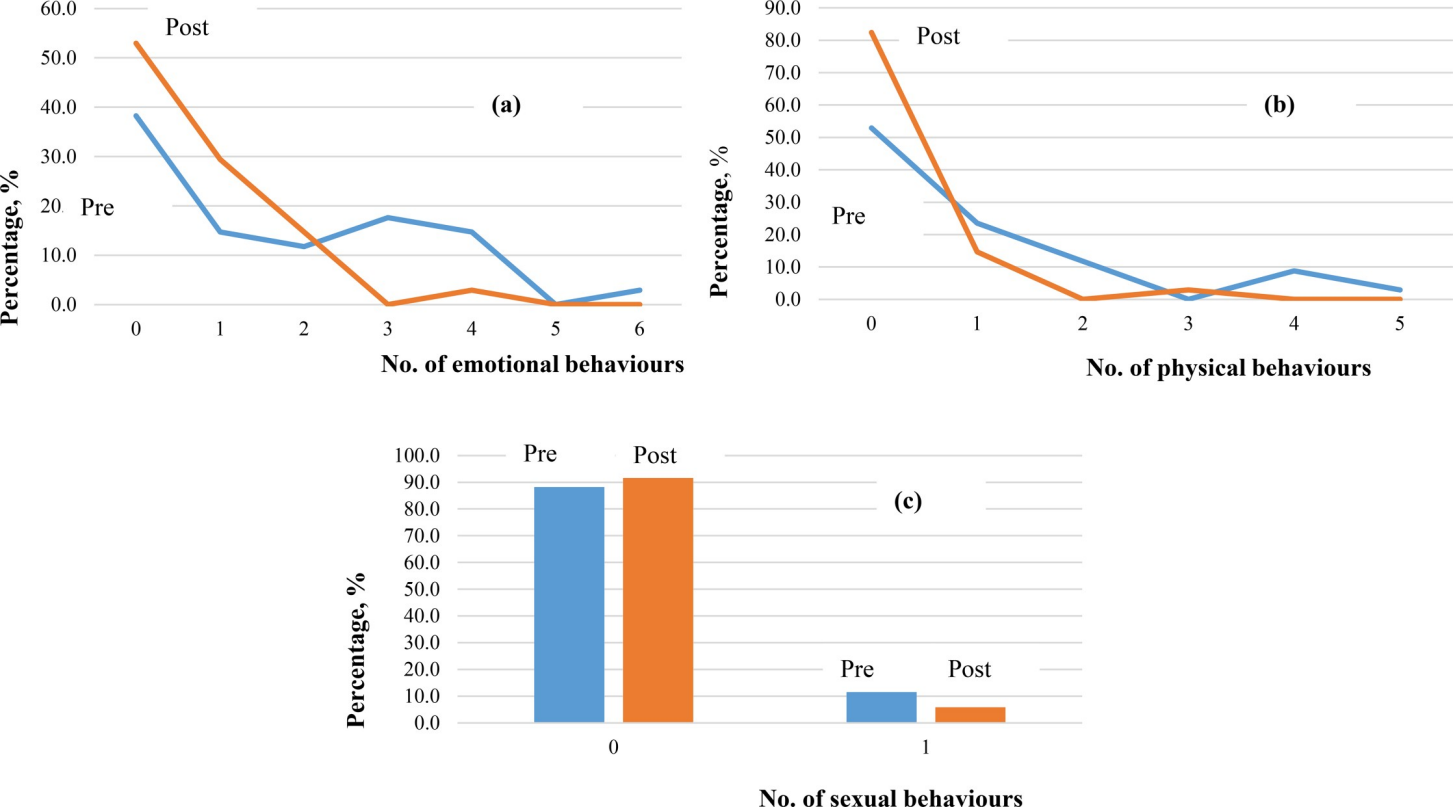
Changes in (a) emotional (b) physical and (c) sexual behaviour score (as defined and shown in Figs 1–3) since completing the programme; using Impact questionnaire at start of programme (‘Pre’) and end of programme (‘Post’).

A total of 34 clients completed the individual IMPACT questionnaire following completion of the RADAR/ADAPT programme. Fig 4 shows the difference in the number of emotional, physical and sexual behaviours before and after the programme. As shown in Fig 4A compared to before the programme, fewer clients had higher emotional scores, and the number of individuals that exhibited two or less behaviours increased. Fig 4B shows the difference in the number of physical behaviours before and after the programme. After the programme, fewer individuals had higher physical scores, and the number of individuals that exhibited 0 behaviours increased. Fig 4C shows the difference in the number of sexual behaviours before and after the programme. Responses to sexual behaviour appeared to be very similar before and after the programme.

#### Police data

Summary statistics are shown in Table 3. Over the monitoring period covered, approximately 28% of clients were linked to crimes (as suspect or charged) after completion. However domestic abuse amounted to less than half of these total crimes (43.2%). Of those who completed, 17.5% of clients were linked to domestic abuse crimes after completion.

**Table 3:** Summary statistics on crime associated with 57 individuals completing the RADAR/ADAPT programme over one year; number shown in brackets.

Percentage of total people who completed the DAPP programme linked to crime after completion, %	28.1 (16)
Percentage of total people who completed the DAPP programme linked to domestic abuse (DA) related crime after completion, %	17.5 (10)
Percentage of total crimes linked after completion that are DA related, %	43.2 (24)
Percentage of total people who completed the DAPP programme linked as suspect/offender to a DA crime after completion, %	62.5 (36)

Note: Follow up period ranged from 10–19 months depending on completion date. Crimes last recorded on 07.06.2018.

#### Qualitative evaluation

Overall two focus groups (n = 12) were conducted with clients (perpetrators) who had either completed, or were close to completing, the programme. A further eight survivors were interviewed during data collection, to explore the extent and nature of any behavioural change observed in their partners, or former partners. Of the survivors interviewed, approximately five were in contact with the partner concerned. Overall there were three major themes, which are outlined in further detail below.

#### Theme 1—Making positive progress

A richer understanding into how the RADAR/ADAPT programme brought about change was gained through focus group discussions. The clients were asked about how their behaviours had been influenced by the programme. Overall, clients indicated that they were making positive progress by attending programme, that the RADAR/ADAPT programme had a big impact compared to other treatments (sub-theme 1), and that both the effort and time spent on the programme were integral to such progress (sub-theme 2). One respondent felt that the programme had enabled him to accept responsibility for his behaviour and suggested that the relationship with his wife had improved as a result of this,

*“Obviously I can’t speak for my wife*, *truthfully*, *but I do touch wood that our relationship has started to actually repair*, *to at least make positive progress*. *Not least because I’ve actually accepted responsibility for what I was actually doing*, *and realising that my behaviour just was completely unacceptable*. *I think almost that has had- I’m trying to think*, *but that has been part of what the course has actually worked on me to actually come to terms with*.”

(Client 4, Focus Group Discussion 1)

Based on survivor feedback, the majority of respondents did not observe or experience any on-going abuse whilst their partners, or former partners, were attending the programme. By contrast, the majority of survivors observed some degree of behavioural change when discussing their relationship,

*“It's changed it for the better*, *obviously because the arguments have stopped*. *It's more like if we've got something to say we'll say it to each other without shouting or building it up inside and not saying anything and then that's when it all gets out of hand*. *He's just been a lot calmer*, *I think it's brilliant*, *it's really helped him*.”

(Interview with survivor, 004)

The same respondent goes on to acknowledge that this change was initiated through an increased sense of self-awareness and understanding of where blame lies,

*“Yes*, *he's definitely*, *his attitude towards life*, *I think*, *has changed a lot more*. *He's actually realised that it was him*, *because I think that's a major step in that sort of self-awareness in domestic violence where they have to realise that it's them*, *because his before was like*, *'Oh*, *it's not me*, *it's you*, *you make me do this*, *you're the one who made me angry*.*' He actually stops and thinks about things before he says them and his just whole perspective*, *the whole*, *I don't know*, *it's weird*! *Definitely a lot calmer though and he's able to talk a bit more now…*.*It weren't that long after*, *to be honest*. *It was probably about a month after the course*.”

(Interview with survivor, 004)

In relation to past behaviour, one survivor identified the *power and control* as an issue in their relationship. Since her partner had been attending the RADAR/ ADAPT programme, however, he had exhibited less controlling behaviours towards the family,

*“It was very much about power and control before*. *Very much he had to be in control of everything and he got very stressed if things weren't as he thought they should be or people didn't do or the children or me or I didn't do things the way he thought…*.”

(Interview with survivor, 002)

On explaining the impact of the change, one client felt that his wife was less fearful of their relationship,

*“She suddenly came out with the phrase*, *'You've really changed*.*' Now*, *I couldn't see that*. *I think I've changed*. *I think*, *in other words*, *I could change*, *but she actually could see*. *To me that was really positive*, *because it has made her*, *because she was very fearful*, *and suddenly she wasn't fearful anymore*. *Suddenly she realised that I was nothing to be afraid of*, *anymore*. *'He's changed enough for me to be able to relax and trust*,*' which was great*, *really*.”

(Client 4, Focus Group Discussion 1)

However, one respondent felt that despite the number of apologies offered by her former partner, certain behavioural traits still persisted, such as anger,

*“Still quite volatile and he has apologised a lot—which is something he's never done before—but then he'll follow that with getting very angry if I don't do what he wants after he's apologised*. *He's still very manipulative with the children and with me*. *Yes*, *he is quite prone to getting very angry*.”

(Interview with survivor, 005)

#### Subtheme 1.1 –The influence of the programme on individuals compared to other treatments

Another example of positive progress driven by this programme was given by one survivor who felt that, compared with the other types of therapy her partner had sought in the past, the RADAR/ADAPT programme had had the biggest influence on her partner’s behaviour.

*“Definitely*, *it was because he's had counselling for years and he saw a psychiatrist once and he's had all sorts*. *He's had CAT*, *cognitive analytical therapy*, *and he's had CBT*, *and things would improve for a while but not to this extent and then they would get worse again*. *Obviously*, *it's fairly short*, *he's only just finished it in December*, *mid-December*, *so obviously it's fairly quite fresh in his mind still*, *so I don't know how*, *whether it will have changed him forever but he is telling me that it's revelation and that he's changed*. *But*, *yes*, *out of everything or*, *over the years*, *out of everything that he's done*, *definitely*, *this has had the biggest impact on him*, *his behaviour*.”

(Interview with survivor, 002)

This view was also corroborated by clients on the programme; a few described how influential the RADAR/ADAPT programme had been on their lives, compared to other therapies.

#### Subtheme 1.2 –Positive outcomes due to engagement with programme

Further to the positive progress made to behaviour, clients’ commitment to the programme also had an impact on their individual circumstances. One client suggested that the effort and time put into the programme was acknowledged by the judge reviewing his custody case. He further described how his relationship with both wife and children had improved,

*“Like yourself*, *I have great shame that I've affected and hurt my children*. *Coming from a place of seeing them supervised six times a year for a couple of hours*, *to seeing them now pretty much when I want*, *or when they want*. *I think it was the reports that [XX] and her colleagues filled in that I could present to social services*, *then they questioned me*, *and examined and interviewed me*. *They made a report*, *and these two reports go to the courts*. *Basically*, *the judge has got something to go on then*, *hasn't he*? *He said to me*, *'You've done the work*. *You've put this effort in*. *We've got a report from the programme*. *We've got a report from social services that interviewed you*,*' and now it's a family on the mend*. *My wife and I are amicable*, *and I'm seeing the children on a*, *basically*, *when they want*, *really*. *I mean*, *they want to go fishing*, *they want to go swimming*. *They're two boys*, *ten and 12*. *I mean*, *they're enjoying it now*, *which is great*, *and they're coming to stay with me at Christmas*.”

(Client 2, Focus Group Discussion 1)

Another client explained that since starting the programme his ex-partner had contacted him, encouraged by his investment in the programme and the positive steps he was making,

*“I've already*, *for the first time in two years*, *I've had an email off my ex-partner because she wants to talk*. *That's literally a massive step*, *the biggest step that I could have ever taken because of being on this course*, *and because we've been consistent in direct contact*. *That's the first step of trust*. *That's the key really*, *I think*. *I hope it's because of this course*, *and perhaps a link with it and the amount of time I've taken to do it*. *If it was six weeks*, *I don't think she'd be convinced*. *I know some of the other guys instantly within… Just by turning up*, *their families*, *they're in relationships now*, *their families have all rallied up round them and supported them because they're making the positive step*. *Is anyone actually in a relationship here*?”

(Client 5, Focus Group Discussion 2)

The reports from the main voluntary sector organisation and partner agencies also facilitated this process; relationships seemed to change, or improve, as a result of attending the programmes and having reports shared with social services,

*“…*.*but because social services are involved*, *they actually presented the reports to my wife*, *and made their own report*. *If not*, *no one would have really explained what I'd been doing*. *Even then*, *even though the report was given to her and explained to her*, *it wasn't until about three months after court*, *and the relationship started to improve*, *and I was seeing the children normally*, *and my wife and I were amicable*.”

(Client 3, Focus Group Discussion 1)

As conveyed in the quote above a positive outcome, or a noticeable change in relationship, could take months.

#### Theme 2—Impact of the children’s module

The children’s module had a powerful influence on clients attending the programme, as represented in this theme. One survivor described the emotional influence of the RADAR/ADAPT programme on her partner, particularly in acknowledging his past behaviours towards their child. One session was singled out, as it had a noticeable impact on her partner’s behaviour towards their daughter,

*“I think he's done really*, *really well*, *to be honest*. *He's surprised me and I think he's surprised himself*. *He's opened up a lot more to me*. *He's been in tears when he's come home*, *I mean I've been with him eight years*, *I've never seen him cry or anything*. *After one session at the course he came home*, *he apologised to my older daughter*. *I think it was the child thing that they had to do and pretend like they were in darkness and asleep and people shouting*, *just put themselves in the child's point of view*, *if you know what I mean*? *I think that really done him in and made him realise and he come home and he was in tears and he apologised*, *like I say*, *to my older daughter and he's apologised to me a million times*. *Yes*, *I think it's a great eye-opener*, *I think it's a wonderful course*.”

(Interview with survivor, 004)

Another client identified the children’s module of the programme as the point in which he noted changes in himself. He reflected on the transgenerational effect of his behaviour, linking to his own adverse childhood experiences, and understanding of how it may impact his own children,

*“Going back to the question you said about*, *'At what point on the programme did you feel a change*?*' For me it was when we covered the children's module*, *because you learn about your own childhood*. *You have a real look at yourself*, *and your own upbringing*, *and the negative behaviours that you took from that*, *albeit learned*. *The facilitators didn't allow you to blame yourself*, *or your parenting*, *but to look at the negative aspects of your own childhood experiences*, *and how that then progressed to your behaviour towards women*, *towards your partners*, *and towards your children*. *Ultimately I could see that I did not want history to repeat itself*, *however it already had started*.“

(Client 4, Focus Group Discussion 1)

In their discussion around change, many focussed their attention on spending time and repairing relationships with children. However one respondent goes on to acknowledge that, even though children may be the motivation, spending time with them may not necessarily be the best thing for them,

*“It's tough getting up every day and trying not to be a dick*, *and if it weren't for the programme*, *then I would be way back*, *but be on the line in terms of even thinking about changing behaviour*. *Like the other guys*, *I've had some small real positives in that we're working towards contact with my children*. *Even back then*, *I think if I'd have had contact with my children two or three years ago*, *when I was still in that mind-set*, *I would have just sort of thought yes*, *I've won*. *I've won*. *I've got an opportunity to see my children*. *As opposed to now I'll think what's best for them*? *That might not be seeing me*, *and that's really hard to deal with*.”

(Client 6, Focus Group Discussion 1)

One survivor also points out that her former partner had focussed more on improving his relationship with his children than their relationship,

*“I don't think he's talked much about the relationship between the two of us; he's focused on children—which is*, *its fine*, *I don't want a relationship with him*. *I just want to support something with the children as they grow up*. *I don't think he's really delved into that very much*.”

(Interview with survivor, 005)

### Theme 3—Concerns around sustaining new behaviours

Finally, clients iterated the importance of sustaining behavioural change, as conveyed through the final theme. To this end, one client considered the potential role of mentors to help sustain change, and to support others to reinforce ideas and behaviours, stating that he was willing to act as a mentor for others who had taken part in the group programme,

*“That's over and done with*, *so perhaps the mental things*, *I'm looking forward to finding out… I can imagine it just drops off completely and my behaviours could just go back to the way they were because there isn't any reinforcement there after the 26 weeks*, *25 weeks*, *so I think that's one of the disadvantages*. *I don't know because I haven't been here*, *so it's difficult for me to say that*. *We know we've got a mentoring scheme*. *I can't imagine what that's like*. *I imagine that's us ringing the mentor rather than them ringing us*, *just to see how things are going*. *I think there may need to be a little bit more… Look into that*, *perhaps the after effects*. *You find out the good and the bad stories afterwards*, *what's going on down the line*. *I've already said that I'd quite happily be a mentor*.”

(Client 6, Focus Group Discussion 1)

For this client, even though he felt his behaviour could revert back to the way it was, he shows self-awareness; even if new behaviours are not fully embedded, awareness around behaviour has somewhat improved. This concern around relapsing behaviours, once the support of the group was removed, was echoed by another client,

*“That's what worries me*. *We're in the terribly vulnerable position that we've already crossed the Rubicon*. *Going back*, *there's no going back*. *You've actually got to actually keep demonstrating it*. *In a way you need a support infrastructure behind it*, *which costs money*, *of course*. *I think this is a fantastic course to go on*, *for me*, *and as a personal thing*, *but I just feel that there is a need to be able to almost have a referral point on*. *To be able to just pick up a phone*, *or have a meeting with somebody*. *Even if it's once a month*, *initially*, *once every six months*.”

(Client 3, Focus Group 2)

## Discussion

This study provides unique evidence on the outcomes of behaviour, reasons for engagement, as well as the types of abuse committed by clients on a community perpetrator programme by using a triangulation design of various data sources including police data. At the individual-level, the data provided through the IMPACT Toolkit showed positive changes in both the physical and emotional behaviours related to domestic abuse after completion of the programme. This is consistent with previous evaluations, which found significant reductions in both physical and sexual violence between baseline and twelve months after programme, taken from the survivors’ perspective [17, 18]. Within the DAPP, survivors also reported improved relationships, particularly in regards to the clients’ relationships with children. The qualitative findings were also supported by police data, showing that just over one in four of the individuals enrolled on the DAPP will go on to commit, or be suspected of, any crime after completion the programme. However, domestic abuse crimes amounted to less than half of these total crimes committed by one in five individuals.

These individual level changes were attributed to the quality and length of the programme. In particular, men felt that the time invested in the programme was necessary to embed new behaviours. This finding is consistent with the evaluation conducted on the Tyneside DVPP, whose programme was *specifically designed* to be long enough to effect real change in men’s abusive behaviour [19]. Furthermore, clients on the RADAR/ADAPT programme cited that both the intensity and peer support were required to facilitate change. In the Mirabel evaluation that examines DVPPs across eleven sites, group work sessions were described as being both informative and useful in promoting change; furthermore the one-size fits all was considered the most acceptable approach [17]. Consistent with findings here, input from both facilitators and other men within the group context enabled this behavioural change process.

As found in previous evaluations, the children’s module was often mentioned by clients, in particular the sessions where clients were asked to put themselves in the position of children living with domestic abuse [17, 19]. The power-control wheel was also frequently mentioned by clients as a useful framework for conceptualising abusive behaviour. The most cited motivating factor for men to join a programme was a willingness to improve relationships, particularly with their children. However, motivation is inherently individual, and there were a number of clients that engaged with a programme who did not have children. As is personal development, with different men being at different stages in their journey towards addressing their own behaviour [20]. According to the transtheoretical model, individuals could be at any stage within the behavioural change process. [21] Some participants had taken steps to initiate change before even starting the programme; for example, by meeting counsellors or therapists. As with other models of behaviour change, change is not a linear process, and some individuals may regress to old behaviours. The RADAR/ADAPT programme is specifically designed to reinforce notions around positive behaviour, moving them towards positive action. However, our results showed that one in five will go on to be a suspect or convicted of a domestic abuse crime after completion. This suggests that, for some individuals, it is difficult to embed new behaviours. Evidence shows that lasting behavioural change can take between six months to five years, supporting the need to maintain new behaviours beyond the end of the programme [22].

### Limitations

When interpreting these findings, it is important to acknowledge the following limitations. Firstly, the outcome data was limited to small numbers (n = 34). The IMPACT Toolkit was relatively new to the DAPP, and therefore it took some time to implement its use, which led to missing data on outcomes. Secondly, the end of intervention questionnaire was only completed by clients of the group, and not by survivors (partners or former partners), due to the practicalities around doing this. It was therefore not possible to triangulate these results with the survivors’ perspective. Some questions in the before/ after questionnaire assessed the insight of the perpetrator, and how his behaviour *affected* his partner. It would therefore be more accurate to evaluate impact of harm through the survivor’s responses. Thirdly, the RADAR/ADAPT programme was specific to the domestic abuse experienced between intimate partners within heterosexual relationships. The implications are that a number of individuals may not find the programmes appropriate to their context, particular those within lesbian gay bisexual transgender queer (LGBTQ) relationships. Finally, the police data included information on those who had completed the programme only. This meant that a comparative analysis, of those who engaged versus those who did not, could not be carried out which would of otherwise provided valuable information.

## Implications

Given that children were a strong motivation for completing a programme, it seemed almost paradoxical that there were no specialist services made available for children within the DAPP model. Consistent with other evaluations on DVPPs, there is a need for a more dedicated support service for children of men on DVPPs, as this is an opportunity to safeguard children. Recent national strategies advocate for early prevention and intervention for domestic abuse, addressing issues *earlier in the cycle* [17–19, 23, 24].

Although there is compelling evidence to suggest that men changed their behaviours following the programme, police reoffending data suggests that, for a minority of individuals, more work is required to fully embed positive behaviours. A mentoring service may support such aims.

To further support evidence on behavioural change, long term outcomes related to survivor harm should be measured for example, through a short questionnaire filled out by current or former partners. As suggested previously, follow-up periods to DVPPs may be too short. [25] Both nationally, and internationally, there is a need to demonstrate the long term outcomes related to programmes, and to triangulate data with survivors’ views. Given that survivor engagement may be poor, shorter versions of questionnaires undertaken by a phone interview may offer a more practical solution [20].
